# Murine cytomegalovirus downregulates ERAAP and induces an unconventional T cell response to self

**DOI:** 10.1016/j.celrep.2023.112317

**Published:** 2023-03-29

**Authors:** Kristina M. Geiger, Michael Manoharan, Rachel Coombs, Kathya Arana, Chan-Su Park, Angus Y. Lee, Nilabh Shastri, Ellen A. Robey, Laurent Coscoy

**Affiliations:** 1 Division of Infectious Diseases and Vaccinology, School of Public Health, University of California, Berkeley, Berkeley, CA 94720, USA; 2 Division of Immunology and Pathogenesis, Department of Molecular and Cell Biology, University of California, Berkeley, Berkeley, CA 94720, USA; 3 Department of Pathology, Johns Hopkins University School of Medicine, Baltimore, MD 21205, USA; 4 Cancer Research Lab, University of California, Berkeley, Berkeley, CA 94720, USA; 5 Lead contact

## Abstract

The endoplasmic reticulum aminopeptidase associated with antigen processing (ERAAP) plays a crucial role in shaping the peptide-major histocompatibility complex (MHC) class I repertoire and maintaining immune surveillance. While murine cytomegalovirus (MCMV) has multiple strategies for manipulating the antigen processing pathway to evade immune responses, the host has also developed ways to counter viral immune evasion. In this study, we find that MCMV modulates ERAAP and induces an interferon γ (IFN-γ)-producing CD8^+^ T cell effector response that targets uninfected ERAAP-deficient cells. We observe that ERAAP downregulation during infection leads to the presentation of the self-peptide FL9 on non-classical Qa-1b, thereby eliciting Qa-1b-restricted QFL T cells to proliferate in the liver and spleen of infected mice. QFL T cells upregulate effector markers upon MCMV infection and are sufficient to reduce viral load after transfer to immunodeficient mice. Our study highlights the consequences of ERAAP dysfunction during viral infection and provides potential targets for anti-viral therapies.

## In brief

Geiger et al. find that MCMV downregulates MHC class I peptide-processing enzyme ERAAP. MCMV-infected mice develop a non-classical QFL T cell response, directed against ERAAP downregulated cells. QFL T cells protect against severe MCMV infection in immunodeficient mice. QFL T cells serve as an unconventional response against MCMV infection.

## Graphical Abstract

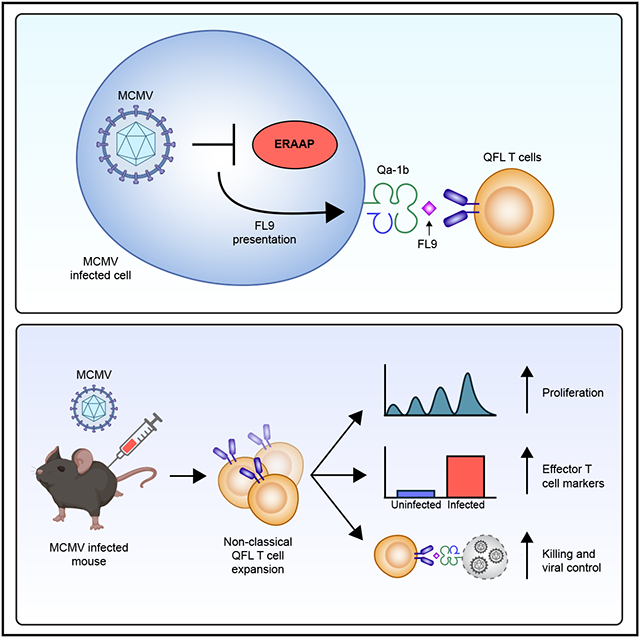

## INTRODUCTION

Viral immune surveillance relies on the antigen processing pathway to generate viral-derived peptides for presentation on major histocompatibility complex (MHC) class I molecules on the surface of infected cells for recognition by cytotoxic CD8^+^ T cells (cytotoxic T lymphocytes [CTLs]).^1^ Peptide and MHC (pMHC) complexes are generated through a series of coordinated steps in which both microbial and self-proteins are initially processed in the cytosol by the proteasome^2,3^ and then transported into the endoplasmic reticulum (ER) through the transporter associated with antigen processing (TAP)^3-5^ and finally trimmed in the ER by ERAAP to the optimal length of 8–10 amino acids for MHC class I binding.^6-9^ To escape CTL detection, many viruses, including human cytomegalovirus (HCMV) and its mouse homolog murine CMV (MCMV), have evolved multiple strategies for targeting MHC class I and the antigen processing pathway.^10-13^ MCMV encodes viral proteins m4, m6, and m152 that target MHC class I through multiple mechanisms, including retaining MHC class I at the ER-Golgi intermediate compartment and redirecting MHC class I for lysosomal degradation.^14-20^ While MHC class I downregulation allows viruses to evade conventional T cell responses, it also activates natural killer (NK) cell responses.^21^ Therefore, MCMV downregulation of MHC class I requires a fine balance between inhibiting conventional CTL responses and activating of NK cell responses. In addition to directly targeting MHC class I,^11,22^ HCMV also targets ERAAP (human ERAP1) mRNA through both miR-UL112-5p and miR-US4-1 to inhibit the processing of HCMV peptides.^23,24^ The impact of viral-mediated ERAAP downregulation on CD8^+^ T cell responses and whether MCMV also targets ERAAP remain unclear.

In the absence of infection, loss of ERAAP leads to an altered display of MHC class I-bound self-peptides.^25-27^ As a result, immunization of wild-type mice with ERAAP-deficient cells induces a CTL response against these pMHC complexes.^28^ Interestingly, a substantial proportion of this response consists of T cells directed against the non-classical MHC class Ib molecule Qa-1b presenting a peptide (FL9) derived from broadly expressed proteins, Fam49 A/B (termed “QFL” T cells). In addition to using a non-classical MHC class Ib-restricting molecule, QFL T cells have other characteristics of unconventional T cells, including the predominant use of a fixed TCR α chain and an antigen-experienced (CD44^+^) phenotype in naive mice.^29,30^ It has been suggested that QFL T cells may monitor cells for ERAAP dysfunction,^28^ but whether QFL T cells play a role during infection remains unknown.

Qa-1b along with its functional homologs HLA-E in humans and Mamu-E in rhesus macaques (collectively called MHC-E) are members of a large family of non-classical MHC class Ib molecules. Unlike classical MHC class Ia molecules, which are polymorphic, bind diverse sets of peptides, and are recognized by diverse αβ T cell receptors (TCRs) on CD8^+^ T cells, non-classical MHC class Ib molecules are non-polymorphic, present a limited set of peptides and non-peptidic ligands, and, in some cases, can be recognized by NK cell receptors as well as some semi-invariant TCRs.^31,32^ In healthy cells, Qa-1b and HLA-E predominantly present conserved leader peptides from MHC class Ia molecules (termed Qdm in mice and VL9 in humans). The Qdm-Qa-1b ligand engages the CD94/NKG2A receptor and provides an inhibitory signal primarily for NK cells but also for CD8^+^ T cells expressing this receptor.^33-35^ Subsequently, it was shown that Qa-1b and HLA-E molecules could also present an array of self and microbial peptides to CD8^+^ T cells when there are defects in the antigen processing pathway.^36-39^ In the context of ERAAP deficiency, Qa-1b binds to a more extensive set of peptides, triggering CD8^+^ T cell responses.^27^ Recent interest in MHC-E has been stimulated by a rhesus macaque CMV (Rh-CMV) vectored vaccine that elicits a broad and protective MHC-E-restricted CD8^+^ T cell response against simian immunodeficiency virus (SIV) infection.^40-47^ Because of the limited poly-morphism of MHC-E and the promising results from the SIV vaccine, unconventional MHC-E-restricted T cells show great potential in vaccine development. However, our limited understanding of how MHC-E-restricted T cell responses are generated and how they provide protection hampers our ability to realize the clinical potential of these responses.

MHC-E-restricted CD8^+^ T cells have been described in both HCMV and MCMV infections.^48,49^ However, whether these T cell responses are shaped by and respond to ERAAP downregulation during infection is not known. In this study, we find that ERAAP protein levels are downregulated in MCMV-infected cells, resulting in a robust immune response that targets uninfected ERAAP knockout cells. MCMV infection *in vivo* stimulated the expansion and effector differentiation of MHC-E-restricted QFL T cells, and QFL T cells were protective in MCMV-infected Rag2/γc knockout mice. QFL T cells therefore detect changes in the antigen processing pathway during MCMV infection and serve as an unconventional immune response against infection.

## RESULTS

### MCMV downregulates ERAAP, leading to the presentation of FL9-Qa-1b

**Figure 1.**
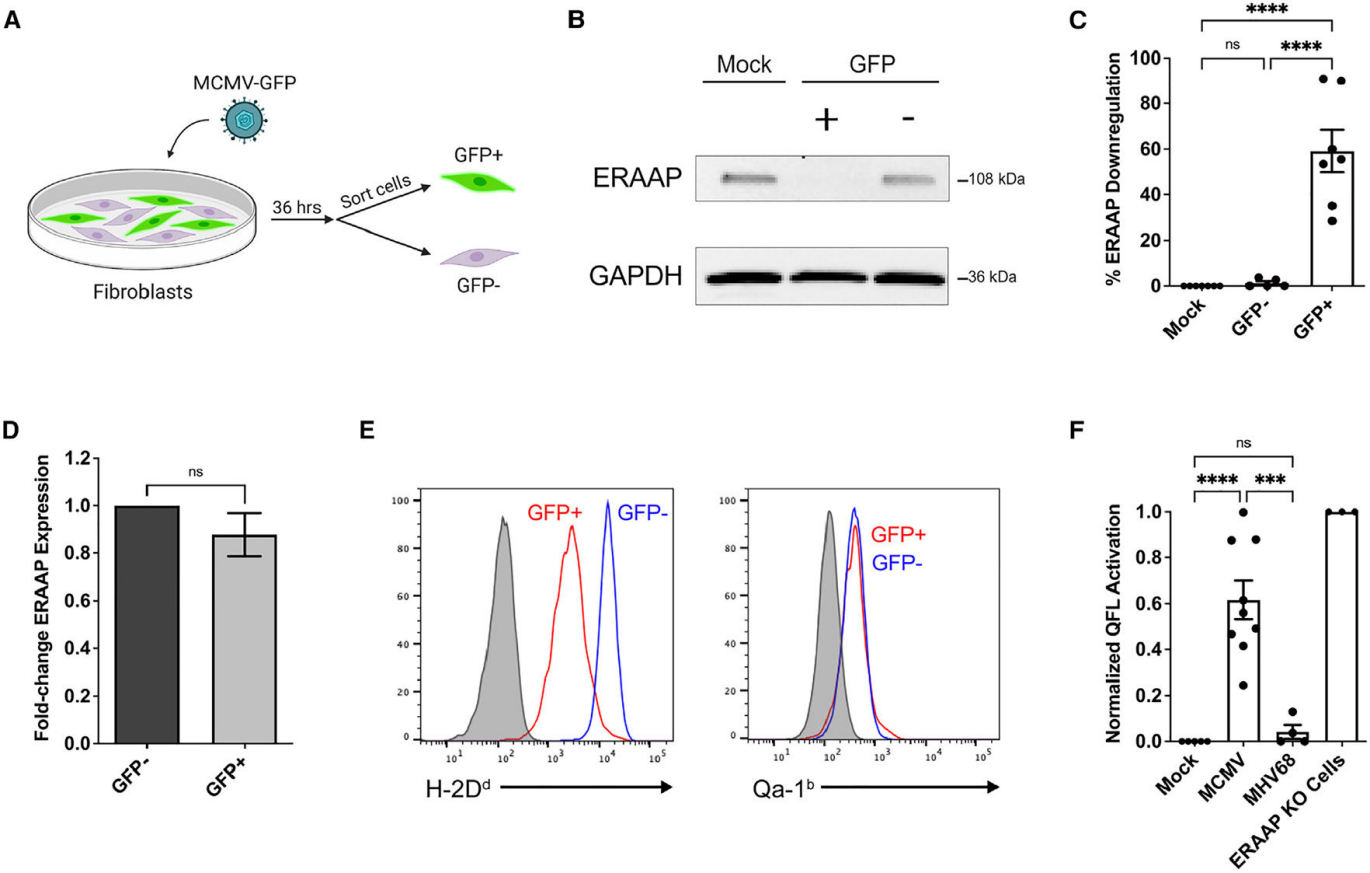
MCMV downregulates ERAAP protein and leads to the presentation of the QFL ligand FL9-Qa-1b (A–D) B6 fibroblast cells were infected with MCMV-GFP (MOI 10), and after 36 h, GFP^+^ and GFP^−^ cells were sorted and assayed by western blot or qRT-PCR. (B) Representative western blot showing ERAAP protein levels in mock-infected, GFP^+^, and GFP^−^ samples, with GAPDH as the loading control. (C) Compilation of percentage of ERAAP downregulation in GFP^+^ and GFP^−^ samples compared with mock-infected cells. ERAAP protein band intensity levels were calculated and normalized to the corresponding GAPDH band intensity level. Percentage of ERAAP downregulation was calculated by comparing normalized ERAAP protein levels in each sample with the mock-infected sample. Data were pooled from five independent experiments and are shown as mean ± SEM. (D) Quantitative real-time PCR analysis of relative ERAAP mRNA levels in GFP^+^ compared with GFP^−^ sorted cells after normalization to GAPDH. Data were pooled from two independent experiments. (E) A macrophage cell line (RAW 264.7) was infected with MCMV (MOI 10) and analyzed by flow cytometry for surface expression of classical MHC H-2D^d^ (left panel) and non-classical MHC Qa-1b (right panel) in gated GFP^−^ and GFP^+^ cells. The experiment is representative of two replicates. Gray histograms represent isotype antibody control. (F) Presentation of the QFL ligand FL9-Qa-1b measuring the lacZ response of QFL-reactive BEko8Z T cell hybridoma cells incubated with mock-infected, MCMV-infected, MHV68-infected, and ERAAP KO fibroblast cells. Infected cells were infected at an MOI 10. The hybridoma response was measured at an OD595 and then normalized to mock-infected samples. The percentage of QFL activation was calculated by comparing the fold change of the normalized response of each sample with the response against ERAAP KO cells. Data were pooled from three independent experiments and are shown as mean ± SEM. Comparisons between samples were made using an ordinary one-way ANOVA multiple comparisons test, *p < 0.0332, **p < 0.0021, ***p < 0.0002, ****p < 0.0001. See also Figures S1-S3.

Herpesviruses target various proteins in the antigen processing pathway to evade immune responses, including the downregulation of ERAP1 mRNA by HCMV.^23,24^ To test whether MCMV also targets ERAAP, we measured ERAAP expression in infected cells. We infected B6 fibroblast cells with a GFP-expressing MCMV strain for 36 h and sorted infected (GFP^+^ cells) and uninfected (GFP^−^ cells) by fluorescence-activated cell sorting (FACS) (Figure 1A). Western blot analysis in each sorted sample revealed decreased ERAAP protein levels of up to 90% in GFP^+^ infected cells compared with GFP^−^ uninfected and mock-infected cells (Figures 1B and 1C). Downregulation of ERAAP protein was also observed upon MCMV infection of additional cell lines, including RAW macrophages and L cells (Figures S1 and S2), indicating the generality of the phenomenon.

HCMV infection leads to the downregulation of ERAP1 mRNA through viral microRNAs miR-US4-1 and miR-UL112-5p. To determine whether MCMV targeted ERAAP by a similar mechanism, we used qRT-PCR to measure and compare ERAAP mRNA levels in FACS-sorted MCMV-infected GFP^+^ and uninfected GFP^−^ cells. We found that ERAAP mRNA levels in GFP^+^ infected cells did not change compared with GFP^−^ uninfected cells (Figure 1D). These data suggest that unlike with HCMV infection, MCMV does not lead to reduced ERAAP mRNA levels and instead inhibits ERAAP at the translational or protein level. A screen of MCMV open reading frames (ORFs) did not point to a single viral gene responsible for ERAAP downregulation, suggesting that multiple viral genes and/or disruption of a host cell pathway might be responsible for ERAAP downregulation in infected cells.

MCMV infection leads to strong MHC class Ia downregulation, but whether Qa-1b levels are impacted is unknown. To address this question, we infected RAW macrophages with MCMV-GFP and assessed the surface levels of both classical H-2D^d^ (as a representative of classical MHC class Ia molecules) and non-classical Qa-1b levels by flow cytometry in GFP^−^ and GFP^+^ cells. Classical H-2D^d^ levels were strongly downregulated in infected GFP^+^ cells, while non-classical Qa-1b levels remained the same compared with GFP^−^ cells (Figure 1E). We also tested other cell lines, such as L cells, and found that Qa-1b levels remained unchanged in infected GFP^+^ versus GFP^−^ uninfected cells (Figure S3). This indicates that MCMV does not alter Qa-1b expression.

Due to the strong downregulation of ERAAP and normal Qa-1b levels observed in MCMV-infected cells, we hypothesized that infected cells would present a peptide repertoire similar to one observed with ERAAP knockout cells. Thus, we investigated whether infected cells presented the QFL ligand (FL9 peptide bound to Qa-1b). To do so, we used an FL9-Qa-1b-reactive T cell hybridoma cell line (BEko8Z) with a TCR-inducible LacZ gene^28^ and L cells expressing Qa-1b as antigen-presenting cells to test the ability of MCMV-infected L cells to activate the T cell hybridomas (Figure 1F). L cells were infected with MCMV for 36 h before incubation with BEko8Z cells for 24 h. We determined BEko8Z activation, as indicated by the induction of LacZ, by measuring the colorimetric cleavage of the β-galactosidase substrate CPRG. BEko8Z cells responded strongly to MCMV-infected L cells and did not respond to L cells infected with a different herpesvirus, MHV68. These results indicate that MCMV infection leads to the presentation of the FL9 peptide on Qa-1b, likely due to viral downregulation of ERAAP protein levels.

### MCMV infection induces CD8^+^ T cell responses that target ERAAP KO cells

**Figure 2.**
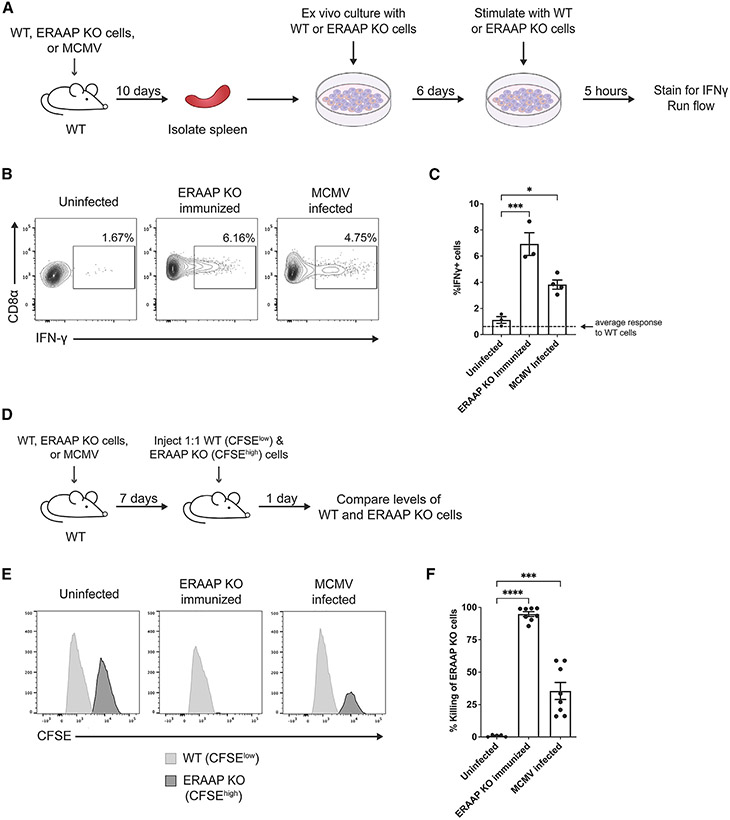
MCMV infection stimulates a response to ERAAP-deficient cells (A–C) C57Bl/6 (WT) mice were infected with 1e6 PFU MCMV (MCMV infected), immunized with ERAAP KO splenocytes (ERAAP KO immunized), or immunized with WT splenocytes (uninfected). At 10 days post-immunization or infection, splenocytes were isolated from mice and stimulated *ex vivo* with irradiated ERAAP KO splenocytes for 6 days. After 6 days, cultured stimulated cells were restimulated *in vitro* with CD4^+^- and CD8^+^-depleted WT or ERAAP KO target splenocytes for 5 h to measure intracellular IFN-γ levels. (B) Representative flow cytometry plots of IFN-γ-producing CD8α^+^ cells from uninfected, ERAAP KO-immunized, and MCMV-infected mice against ERAAP KO target spleen cells. (C) Compiled data of the percentage of CD8α^+^IFN-γ^+^ cells in each group stimulated against ERAAP KO cells. The dotted line represents the average percentage of CD8α^+^IFN-γ^+^ cells against WT target spleen cells. Data were pooled from two independent experiments, with 1–2 samples for each condition per experiment, and are shown as mean ± SEM. (D) C57Bl/6 (WT) mice were infected with MCMV, immunized with ERAAP KO, or immunized with WT splenocytes (uninfected). At 7 days post-infection, mice were injected with splenocytes from WT or ERAAP KO mice that had been CFSE labeled with 0.5 (CFSE^low^) and 5 μM (CFSE^high^), respectively. (E) Representative flow cytometry plots for CFSE levels of transferred WT and ERAAP KO cells, showing the killing of CFSE^high^ ERAAP KO targets in ERAAP KO-immunized and MCMV-infected mice. (F) Compiled data for the percentage of killing of ERAAP KO target cells. The percentage of killing was calculated as follows: 1 – (%CFSE^hi^ sample/%CFSE^lo^ sample)/(%CFSE^hi^ uninfected/%CFSE^lo^ uninfected) × 100. Data were pooled from two independent experiments and are shown as mean ± SEM. Comparisons between samples were made using an ordinary one-way ANOVA multiple comparisons test, *p < 0.0332, **p < 0.0021, ***p < 0.0002, ****p < 0.0001.

If ERAAP downregulation occurred *in vivo* during MCMV infection, we might expect MCMV-infected mice to generate an effector CD8^+^ T cell response against ERAAP knockout (KO) cells, similar to that observed upon immunization of mice with ERAAP KO splenocytes.^28,50^ To examine this question, we infected mice with MCMV and, 10 days later, isolated splenocytes from infected mice and restimulated them *ex vivo* with wild-type (WT) or ERAAP KO spleen cells (Figure 2A). We intracellularly stained and analyzed samples by flow cytometry to measure levels of interferon γ (IFN-γ)^+^ CD8^+^ T cells. Our experiments indicate that CD8^+^ T cells from MCMV-infected mice could mount a strong IFN-γ response against ERAAP KO cells. About 4% of CD8^+^ T cells in MCMV-infected mice induced IFN-γ against ERAAP KO cells, while no IFN-γ response was seen against WT cells (Figures 2B and 2C). This was comparable to the response of CD8^+^ T cells from WT mice immunized with ERAAP KO spleen cells (ERAAP KO immunized) where 7% of CD8^+^ T cells induced IFN-γ^+^ against ERAAP KO cells. No IFN-γ^+^ response was seen in control WT mice immunized with WT spleen cells (uninfected).

We then assessed the ability of the immune response elicited in MCMV-infected mice to target and specifically lyse ERAAP KO spleen cells using an *in vivo* killing assay (Figure 2D). WT mice were infected with MCMV for 7 days and then injected with a 1:1 mixture of splenocytes from WT and ERAAP KO mice labeled with two different concentrations of proliferation dye CFSE. After 24 h, the proportions of WT versus ERAAP KO populations were compared to measure the percentage of killing in infected mice. During this 24 h period, we did not observe any cell proliferation of CFSE-labeled cells, as this would have translated into separate dilution peaks in the labeled cells. Labeled target cells were also injected into uninfected mice immunized with WT cells or immunized with ERAAP KO cells (ERAAP KO immunized) as negative and positive controls, respectively. As predicted, MCMV infection induced a cytotoxic T cell response that efficiently targeted up to 60% of ERAAP KO target cells (Figures 2E and 2F). ERAAP KO-immunized mice had a more robust response, resulting in an average of 94% killing of ERAAP KO cells. We conclude that ERAAP downregulation during MCMV infection elicits a potent immune response that specifically induces IFN-γ against, and eliminates, ERAAP KO cells *in vivo.*

### QFL T cells proliferate in response to MCMV infection

**Figure 3.**
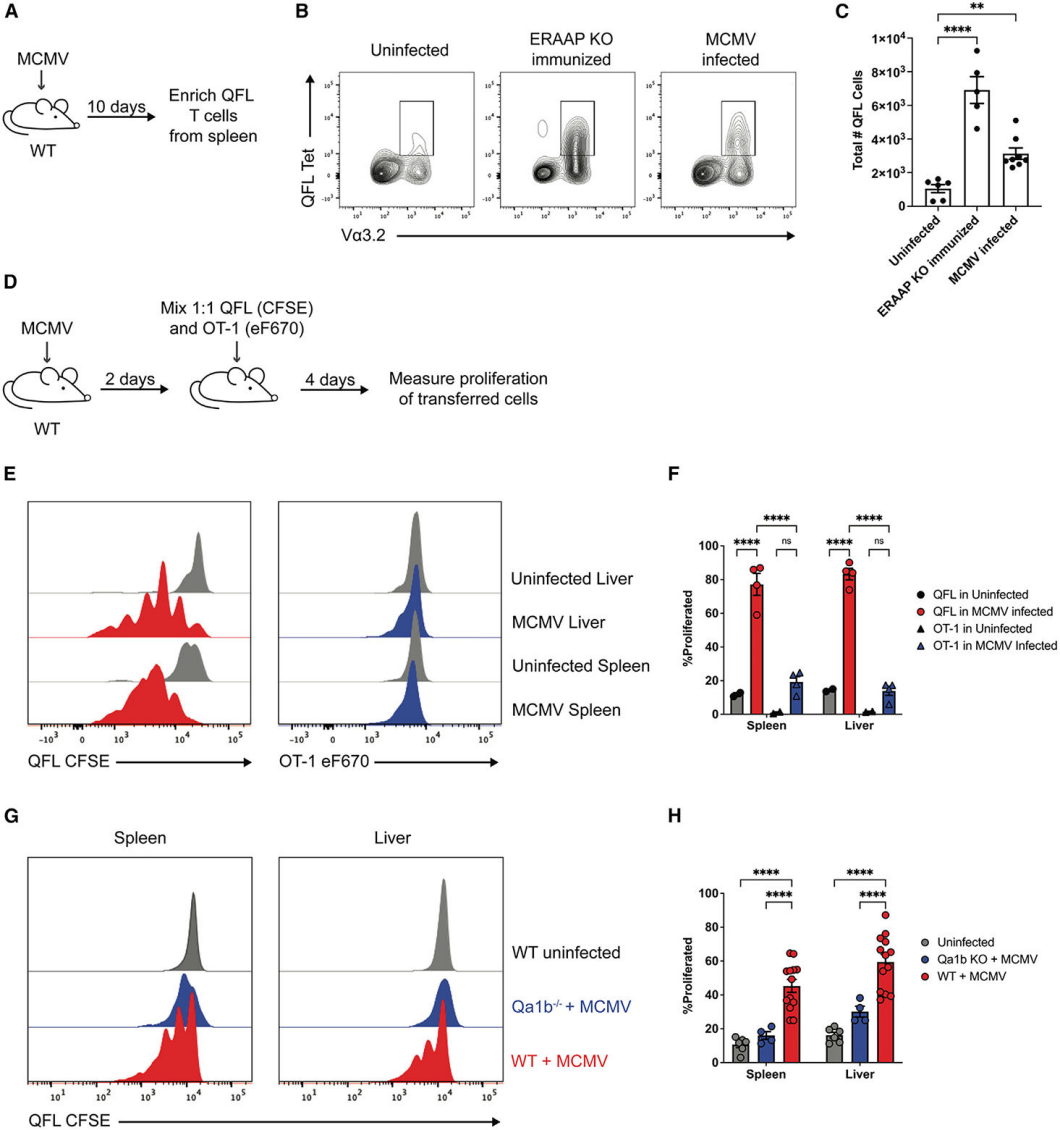
QFL T cells expand and proliferate during MCMV infection (A–C) WT mice were uninfected, immunized with ERAAP KO cells (ERAAP KO immunized), or infected with 1e6 PFU MCMV (MCMV infected), and QFL T cells were stained with FL9-Qa-1b tetramers and enriched from the spleen 10 days post-infection. (B) Representative flow cytometry plots showing QFL expansion by QFL tetramer and Va3.2 expression in each group. (C) Compiled data of the total number of QFL T cells enriched from the spleen in each group over three independent experiments. Data are shown as mean ± SEM. (D–F) WT mice were uninfected or MCMV infected. Two days post-infection, QFL cells (CFSE) and OT-1 cells (eF670) were labeled, mixed 1:1, and transferred intravenously into recipient mice. Four days post-transfer, the proliferation of both T cell subsets was measured in the liver and spleen. (E) Representative flow cytometry plots of QFL (left panel) and OT-1 (right panel) cell proliferation in the spleen and liver of uninfected and MCMV-infected mice. (F) Compiled data from two independent experiments, with 1-2 samples for each condition per experiment, on the percentage of proliferated QFL and OT-1 cells in the spleen and liver of uninfected and MCMV-infected mice. Data are shown as mean ± SEM. (G) Representative flow cytometry plots of levels of proliferation of transferred QFL T cells into WT uninfected, Qa-1b KO MCMV-infected, and WT MCMV-infected mice. WT and Qa-1b KO mice were infected with MCMV. Two days post-infection, QFL cells (CFSE) were labeled and transferred intravenously into recipient mice. Four days post-transfer, proliferation was measured in the spleen (left panel) and liver (right panel). (H) Compiled data of the percentage of proliferated QFL cells in the spleen and liver of WT uninfected, Qa-1b KO MCMV-infected, and WT MCMV-infected mice. Data were pooled over three independent experiments and are shown as mean ± SEM. Comparisons between samples were made using a standard two-way ANOVA multiple comparisons test, *p < 0.0332, **p < 0.0021, ***p < 0.0002, ****p < 0.0001.

QFL T cells are found at a relatively high frequency and exhibit a mostly CD44+ phenotype in WT mice but expand further and acquire effector activity upon immunization with ERAAP-deficient splenocytes.^28^ We investigated whether QFL T cells expanded during MCMV infection *in vivo* in response to ERAAP downregulation by the virus. We infected mice with MCMV for 10 days and determined the number of splenic QFL T cells using tetramer enrichment and flow cytometry (Figure 3A). Uninfected mice immunized with WT cells or immunized with ERAAP KO cells (ERAAP KO immunized) served as negative and positive controls, respectively. Our results revealed that MCMV-infected mice had a 3-fold increase in the total number of QFL T cells in the spleen compared with uninfected mice. Specifically, MCMV-infected mice had an average of 3,100 total QFL T cells in the spleen. In comparison, uninfected mice had an average of 1,000 total QFL T cells in the spleen, consistent with previous studies (Figures 3B and 3C).^28^ In addition, all tetramer^+^ QFL T cells expressed the Vα3.2 chain, as previously shown.^30^

To measure *in vivo* presentation of the QFL ligand (FL9-Qa-1b), we generated a transgenic mouse line harboring the rearranged TCR α and β genes from the FL9-Qa-1b-reactive Beko8Z hybridoma (QFL TCR transgenic mice). We transferred CFSE-labeled splenocytes from QFL TCR transgenic mice into WT mice infected 2 days prior with MCMV. We measured the proliferation of transferred QFL T cells in different tissues 4 days after transfer (Figure 3D). We focused on the liver and spleen of infected mice, which are known sites of acute MCMV infection.^51^ To confirm that QFL proliferation was due specifically to FL9-Qa-1b presentation and not due to a non-specific inflammatory response to viral infection, we also co-transferred eF670-labeled transgenic OT-1 T cells, specific for the OVA-K^b^ ligand, which is not present in our system.^52,53^ We found that in MCMV-infected mice, an average of 77% of transferred QFL T cells proliferated in the spleen, and 83% of QFL T cells proliferated in the liver (Figures 3E and 3F). OT-1 T cells proliferated at significantly lower levels, with about 19% and 13% of transferred OT-1 cells proliferating in the spleen and liver, respectively. In addition, QFL T cell proliferation induced by MCMV infection was significantly reduced in Qa-1b-deficient (*Qa-1b*^−/−^) MCMV-infected mice (Figures 3G and 3H), further confirming the specificity of this response. These data suggest that QFL T cells encounter their high-affinity ligand in the liver and spleen of MCMV-infected mice, leading to their proliferation and expansion. However, we cannot completely exclude the possibility of these cells proliferating in other tissues and migrating to the liver and spleen.

### QFL T cells acquire an effector phenotype upon MCMV infection

**Figure 4.**
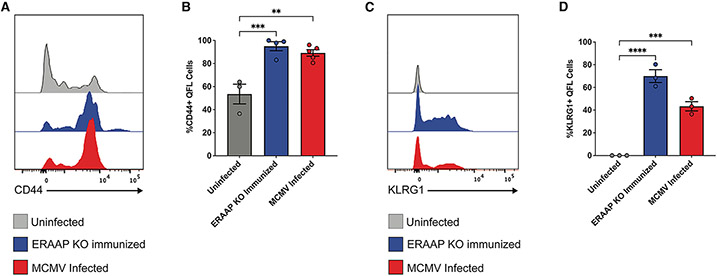
QFL T cells upregulate effector markers CD44 and KLRG1 in MCMV-infected mice (A and B) WT mice were uninfected, ERAAP KO immunized, or MCMV infected (1e6 PFU). QFL T cells were enriched from the spleen of each mouse, and CD44 and KLRG1 expression was analyzed. (A) Representative flow cytometry plots for CD44 expression in QFL T cells. (B) Compiled data of percentage of CD44^+^ QFL T cells for each group. Data are shown as mean ± SEM. (C) Representative flow cytometry plots for KLRG1 expression in QFL T cells. (D) Compiled data of percentage of KLRG1^+^ QFL T cells for each group. Data from (B) and (C) were pooled over two independent experiments, with 1–3 samples for each condition per experiment, and are shown as mean ± SEM. Comparisons between groups were made using an ordinary one-way ANOVA multiple comparisons test, *p < 0.0332, **p < 0.0021, ***p < 0.0002, ****p < 0.0001.

We next investigated how MCMV infection impacted the activation phenotype of QFL T cells. To characterize the effector phenotype and potential anti-viral role of these QFL T cells during MCMV infection, we examined the expression of KLRG1, an effector marker expressed on cytotoxic, proliferative, and virus-specific T cells,^54-56^ along with CD44, a marker of antigen experience, on QFL T cells in mice 10 days after infection with MCMV (Figure 4). As hypothesized, QFL T cells further upregulated CD44 from 53% in uninfected to 89% in MCMV-infected mice (Figures 4A and 4B). QFL T cells also upregulated KLRG1 from 0% in uninfected to 43% in MCMV-infected mice (Figures 4C and 4D). CD44 and KLRG1 levels on QFL T cells from MCMV-infected mice were comparable to the levels found in ERAAP KO-immunized mice. Thus, QFL T cells expand and differentiate into effector and cytotoxic T cells during MCMV infection.

### QFL T cells protect against severe MCMV infection

**Figure 5.**
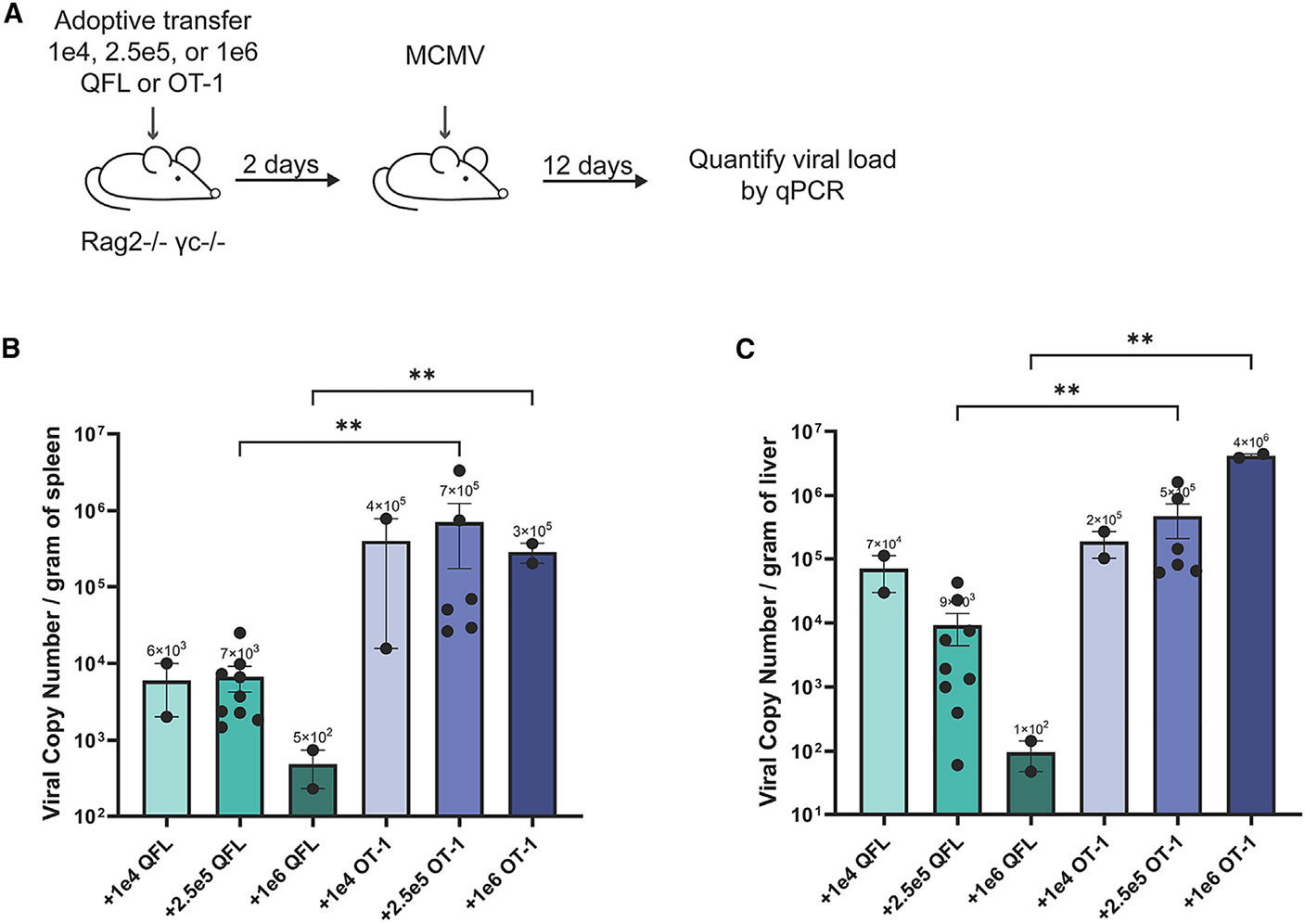
QFL T cells protect against severe MCMV infection (A–C) 1e4, 2.5e5, or 1 e6 QFL or OT-1 T cells from TCR transgenic mice were adoptively transferred into Rag2/γc KO mice. Two days post-transfer, mice were infected with 1e6 PFU MCMV. Twelve days post-infection, the liver and spleen were isolated from infected mice, and MCMV titers were determined by qPCR. (B and C) Compiled data of viral titers in the spleen (B) or liver (C) of infected Rag2/γc KO mice adoptively transferred with different dosages of QFL or OT-1 cells. Data are shown as mean ± SEM, with dots representing individual mice. Data for mice receiving 2.5e5 QFL or OT-1 cells were from two independent experiments, and data for mice receiving 1e4 and 1e6 QFL or OT-1 cells were from one experiment. Comparisons between samples were made using an ANOVA multiple comparisons test, **p < 0.0021. Only significant differences are indicated.

Due to the expansion and effector differentiation of QFL T cells during MCMV infection, we predicted that QFL T cells could protect against MCMV infection. To test this, we adoptively transferred different doses of transgenic QFL T cells (1e4, 2.5e5, or 1e6 QFL T cells) into immunodeficient Rag2/γc KO mice, which lack functional T, B, and NK cells. Rag2/γc KO mice were infected with MCMV 2 days posttransfer. The viral load was assessed 12 days after infection in the spleen and liver (Figure 5A). As a control, we transferred matched dosages of transgenic OT-1 T cells (specific for ovalbumin [OVA], which is not present in our system) into Rag2/γc KO mice to compare the anti-viral response of a non-specific transgenic T cell response with the QFL T cell response. qPCR analysis of the MCMV titers in the spleen and liver of infected QFL recipient mice showed a significant reduction of viral load in both tissues for mice that received 2.5e5 and 1e6 QFL cells compared with matched OT-1 recipients. Immunodeficient mice that received 2.5e5 QFL T cells had about a 2-log reduction in MCMV titers in the spleen (Figure 5B) and liver (Figure 5C) compared with the OT-1 recipient mice. Immunodeficient mice that received 1e6 QFL T cells had an even more substantial reduction of MCMV viral tiers, with a 2.8-log reduction of titers in the spleen and a 4.6-log reduction of titers in the liver. No significant difference in titers was seen for mice that received 1e4 QFL T cells versus 1e4 OT-1 cells. In sum, mice that received 2.5e5 or 1e6 QFL T cells had a significant reduction in MCMV titers in both tissues, and the difference between QFL versus OT-1 protection is more prominent in the liver. Therefore, QFL T cells effectively reduce MCMV titers in Rag2/γc KO mice.

## DISCUSSION

Viruses and their hosts have co-evolved for millions of years, shaping viral immune evasion strategies and compensatory immune responses from the host. For example, CMV downregulates MHC class Ia molecules to avoid CTL attack, leading to enhanced recognition by NK cells. Here, we show that MCMV also triggers downregulation of the MHC class I peptide-processing enzyme ERAAP in infected cells. Given that loss of ERAAP leads to presentation of distinct MCMV peptides,^57,58^ and given that conventional anti-MCMV T cells are primarily primed through cross-presentation by uninfected dendritic cells with normal levels of MHC class Ia and ERAAP,^59,60^ ERAAP downregulation could promote partial viral evasion of effector CTLs by altering peptide display on target cells harboring the virus (Figure S4, steps 1–3). However, we also show that mice infected with MCMV develop an unconventional CTL response directed against ERAAP-deficient cells, likely representing a counter response by the immune system (Figure S4, step 4). Thus, both viral downmodulation of ERAAP and the immune response to the loss of ERAAP are part of the layered interplay between MCMV and the host immune system.

Previous studies identified an unconventional T cell response to a self-peptide presented by Qa-1b (QFL T cells) that makes up a prominent part of the T cell response induced by immunization of WT mice with ERAAP-deficient cells.^28^ QFL T cells are relatively abundant and display an antigen-experienced phenotype in naive mice,^28^ suggesting that they are poised to respond rapidly upon challenge. This is in line with the modest expansion and rapid effector differentiation of QFL T cells observed upon MCMV infection in the current study. MCMV also elicits a robust conventional CD8^+^ T cell response that is initially broad but that, over time, becomes dominated by the expansion of a few MCMV epitopes as the infection progress into latency, a phenomenon known as memory inflation.^61^ Considering T cell numbers, it seems likely that QFL T cell protection is most relevant in the early phase of infection, prior to the massive expansion of conventional CD8^+^ T cells, and perhaps overlapping in time with NK cell responses. QFL T cells may contribute to the early anti-viral response by direct killing of infected cells and production of cytokines or may play a more regulatory role.^62^ For example, QFL T cells could limit the extent of the conventional T cell response, as it has been shown for NK cells and NKT cells.^63-67^

It is interesting to consider what cells might serve as antigen-presenting cells (APCs) for QFL T cells during infection. Given the high precursor frequency and antigen-experienced phenotype of QFL T cells in uninfected mice, they may not require priming by an activated dendritic cell (DC) as do naive T cells. In addition, since Qa-1b and Fam49 A/B are broadly expressed, QFL T cells could potentially expand and differentiate upon encounter with any virally infected cell. For example, in the liver, where we observed QFL T cell proliferation in infected mice, the virus can replicate in liver sinusoidal cells, including endothelial cells and Kupffer cells.^68-70^ In addition to expanding and promoting effector functions of QFL T cells, virally infected cells may also serve as targets for killing, helping to explain the protective effect of QFL T cells on viral control reported here.

Protective Qa-1b-restricted CD8^+^ T cell responses have been described in several infection models, including *Mycobacterium tuberculosis, Listeria monocytogenes, Salmonella typhimurium,* and MCMV.^36,39,48,71,72^ While thus far these responses appeared to be directed against microbial peptides, we show here that a response to self-peptides can form a part of these protective responses. In that regard, the downregulation of ERAAP could serve as a kind of “danger signal,” alerting the host to disruption of cellular homeostasis by altering peptide display on MHC-E. T cells reactive to this form of altered self may not be overtly self-reactive in healthy animals due to the predominant presentation of Qdm peptides and the low surface expression level of MHC-E on normal cells. However, they may nevertheless acquire a pre-activated/memory phenotype due to low-level or infrequent encounters with altered self on abnormal cells and thus may respond more rapidly upon infection. It is tempting to speculate that such a pre-formed T cell response may enhance other anti-viral non-classical T cell responses, including those directed against viral peptides. Of particular interest is the broad and protective CD8^+^ response restricted to MHC-E elicited by an Rh-CMV vectored SIV vaccine.^40-47^ While it is not yet known if Rh-CMV downregulates ERAAP, a QFL-like response to self may contribute to the potent anti-viral MHC-E-restricted response in this setting.

In healthy cells, MHC-E molecules are predominantly loaded with a single peptide derived from the leader peptides of MHC1a molecules (called Qdm in mice and VL9 in primates), and this complex inhibits NK cells via the inhibitory receptor NKG2A. Qdm peptide display is reduced, but not absent, on ERAAP KO cells,^27,73^ suggesting that ERAAP downregulation by viruses such as HCMV^23,24^ and MCMV (current study) may not strongly sensitize infected cells to NK cell killing. In contrast, downregulation of TAP, which occurs in HCMV- and Rh-CMV-infected cells, leads to a loss of the VL9-MHC-E complex, which is countered by expression of virally encoded mimics of VL9.^19,46^ Interestingly, MCMV encodes an MHC class I-like viral decoy m157^74,75^ that activates Ly49H^+^ NK cells in C57Bl/6 mice. This dominant NK cell response would likely mask any potential increase in NKG2A-initiated NK cell responses due to ERAAP downregulation. With this, it is likely that the major immune response induced by ERAAP downregulation in MCMV-infected cells is the activation of QFL T cells.

HCMV is ubiquitous worldwide, with 80%–90% of the global population being seropositive, and the virus is the leading infectious cause of congenital neurological diseases and deafness.^76-78^ Our work applies to HCMV models and suggests there could be significant value in identifying an HLA-E-restricted QFL-like T cell response that detects ERAAP downregulation and contributes to protection against HCMV infection. Our findings can also be applied to other infectious diseases, such as tuberculosis and HIV, which also induce CTL responses restricted to non-classical MHC. In addition, several cancer models have also been shown to have attenuated ERAAP levels,^79^ leading to a presentation of immunogenic tumor antigens and enhanced anti-tumor immunity.^80^ The role of QFL T cells in different infectious disease and anti-tumor models remains to be determined but is a promising path to explore. This work provides avenues for anti-viral immunotherapy approaches to detect and eliminate both infected and cancerous cells with ERAAP dysfunction. For example, a vaccine designed to target a self-peptide presented in the context of ERAAP deficiency by non-classical Qa-1b/HLA-E could be used universally to stimulate similar CTL responses across individuals, despite potential patient-to-patient differences in MHC class I genotype. Other approaches, such as monoclonal antibody therapies that target altered self-peptides, may also be an exciting avenue to explore.

### Limitations of the study

In this study, we assessed the protective capacity of QFL T cells in a Rag2/γc ko transfer model that is devoid of NK and conventional T cell responses. Thus, while we demonstrate that QFL T cells can provide protection, further studies will be needed to access their contribution to anti-viral protection in WT mice.

While the *in vivo* killing of ERAAP KO cells in MCMV-infected mice (Figures 2E and 2F) is consistent with the *in vivo* activation of QFL T cells in infected mice (Figure 3), it is unlikely that QFL T cells account for all of the killing activity observed. For example, other unconventional T cell responses to loss of ERAAP, as well as NK cells, could contribute to this *in vivo* killing activity. Further studies will be required to identify all of the immune components that target ERAAP-deficient cells in MCMV-infected mice.

In this study, we access the expansion of QFL T cells upon MCMV infection (approximately 3-fold) by using QFL tetramer enrichment and staining of splenocytes from infected and uninfected mice (Figures 3A-3C). While at first glance this modest expansion appears at odds with the extensive proliferation of transgenic QFL T cells observed upon transfer into infected mice (Figures 3E and 3F), the transfer assay does not account for T cell migration and survival. Further studies will be needed to determine the proliferation and survival rate of endogenous QFL T cells during infection.

## STAR★METHODS

### RESOURCE AVAILABILITY

#### Lead contact

Further information and requests for resources and reagents should be directed to and will be fulfilled by the lead contact, Laurent Coscoy (lcoscoy@berkeley.edu).

#### Materials availability

QFL transgenic mice are available upon request.

#### Data and code availability

- Original western blot images have been deposited at Mendeley Data and are publicly available as of the date of publication. Mendeley Data: https://doi.org/10.17632/jxdrtfnsgy.1.
- This paper does not report original code.
- Any additional information required to reanalyze the data reported in this paper is available from the lead contact upon request.

### EXPERIMENTAL MODEL AND SUBJECT DETAILS

#### Animal models

WT C57Bl/6 mice were purchased from the Jackson Laboratory. The generation of ERAAP-KO mice has been described.^83^ H2-T23 Qa-1b KO mice were generated in the laboratory of H. Cantor (Harvard University) and were the kind gift of E. Engleman (Stanford University). Rag2/OT-I transgenic knockout mice and Rag2/Il2rg Double Knockout mice were purchased from Taconic. All mouse experiments were done with the approval of the Institutional Animal Care and Use Committee of the University of California, Berkeley. All experiments were done using 7-10 week old mice with roughly equal proportions of male and female mice in infection cohorts.

#### Cell lines

B6 fibroblasts were generated as previously described.^83^ Qa-1b expressing L cells were generated as previously described.^28^ NIH 3T3 (ATC#CRL-1658) and RAW 264.7 mouse macrophages (ATCC TIB-71) were purchased from ATCC. QFL hybridomas (BEko8Z cells) were established in the Shastri lab as described.^28^ B6 fibroblasts and splenocytes were cultured in complete RPMI (cRPMI) with 10% FBS (Invitrogen, Carlsbad CA), 100 U/ml Penicillin/Streptomycin (Invitrogen), 2mM L-Glutamine, and 50 μM 2-ME. NIH 3T3 and RAW 264.7 cells were cultured in complete DMEM with 10% FBS (Invitrogen, Carlsbad CA) and 100 U/ml Penicillin/Streptomycin (Invitrogen). All cell lines were maintained at 37°C, 5% CO_2_, and 95% humidity.

### METHOD DETAILS

#### Mice, immunizations, and infections

Uninfected mice were immunized with 2e7 WT splenocytes, and ERAAP-KO immunized mice were immunized with 2e7 ERAAP-KO splenocytes intraperitoneally. All infected mice were infected with 1e6 PFU of WT MCMV intraperitoneally.

#### Infection of cell lines

Cell lines were infected at an MOI 10 for all *in vitro* MCMV infection experiments.

#### Generation of QFL transgenic mice

The QFL transgenic mice were generated on the B6 background in the Cancer Research Laboratory Gene Targeting Facility at UC Berkeley under standard procedures. The QFL TCR alpha and beta chain sequences were previously identified and amplified from the genomic DNA of QFL BEko8Z hybridoma cells.^28,30^ The TRAV9D-3 TCR alpha chain was cloned with the forward primer (5’ AAAACCCGGGCCAAGGCTCAGCCATGCTCCTGG) with an added XmaI cutting site at the 5’ end of the DNA sequence and a reverse primer for TRAJ21 (5’ AAAAGCGGCCGCATACAACATTGGACAAGGATCCAAGCTAAAGAGAACTC) with an added Not1 cutting site at the 5’ end of the DNA sequence. The TCR beta chain was cloned with the forward primer (5’ AAAACTCGAG CCCGTCTGGAGCCTGATTCCA) with an added Xho1 cutting site at the 5’ end of the DNA and a reverse primer for TRBJ2-7 (5’ AAAACCGCGGGGGACCCAGGAATTTGGGTGGA) with a SacII cutting site flanking the 5’ end of the DNA sequence. The cloned TCR alpha chain was cloned into the pTa cassette vector using the Xmal and Not1 restriction sites, while the TCR beta chains were cloned into the pTb cassette vector using Xhol and SacII restriction sites.^86^ The ampicillin resistance gene was removed from the pTa and pTb cassettes by digestion with EarI. The QFL mice were maintained on the B6 background and bred once with B6 Ly5.1 mice to generate (QFLTgxB6 Ly5.1/2) background mice for use in experiments. Founder mice were identified by flow cytometry and PCR genotyping of tail genomic DNA using the primers mentioned above.

#### QFL hybridoma BEko8Z assay

Hybridoma cells were used in a LacZ assay as described (Sanderson and Shastri, 1994). Briefly, fibroblasts expressing Qa-1b were plated at 20,000 cells/well in a 96-well plate and infected with MCMV or MHV68 at an MOI 10 for 24 hours before the addition of 1e5 QFL hybridoma cells. After 16 hours, cells were spun down at 1500rpm for 5 minutes before the addition of a fluorescent β-galactosidase substrate. The absorbance of each well was examined 12 hours after incubation at a wavelength of 595nm.

#### Virus production and propagation

VR-194 (Smith) strain of MCMV was obtained from The American Type Culture Collection. Smith strain MCMV-GFP was from the Hamilton lab (Duke University, Durham, NC).^81^ MHV68 was obtained from the laboratory of San Speck (Emory vaccine center, The Emory Vaccine Center, Atlanta, Georgia). NIH 3T3 cells were used to produce and titer the virus by TCID50 as previously described.^87^

#### Western blot

B6 fibroblasts were infected with MCMV-GFP for 36 hours at an MOI 10 and then sorted by FACS. Mock infected and GFP+/GFP− sorted cells were lysed in protein lysis buffer 25 mM Tris-HCl (pH 7.6), 150 mM NaCl, 0.1% SDS, 0.5% sodium deoxycholic, and complete EDTA-free protease inhibitors (Roche) for 30 minutes on ice. After, samples were centrifuged at 20,000xg for 10 min at 4°C to clarify the lysate. Lysates were separated by SDS-PAGE, and western blotted with rabbit anti-ERAAP (1:1000) or mouse anti-GAPDH (1:5000; Abcam, ab8245). Rabbit anti-ERAAP was produced in the Shastri lab.

#### RT-qPCR and qPCR

qPCR was conducted using an ABI7300 RT-qPCR System with the following protocol: 95°C dissociation step for 15 sec, 60°C amplification step for 1 min, repeated for 40 cycles. The Applied Biosystems 7300 SDS software was used to calculate Cq values. Each sample was done in triplicate and averaged. DNA and RNA extractions were done as previously described.^87^ Mouse and viral DNA were isolated from mouse tissue using the Qiagen DNeasy Blood and Tissue Kit (Qiagen). iTAQ universal Syber Green supermix (Invitrogen) and 300uM of MCMV Gb or GAPDH primers were mixed with isolated DNA for qPCR analysis. Absolute viral copy numbers were extrapolated using a standard curve of known quantities of purified MCMV BAC. RNA was extracted from cells using Trizol (Invitrogen). Genomic DNA was removed with 0.002 U of DNase I (Thermo). cDNA was synthesized using 1 ug of RNA reverse transcribed for 50 min at 42°C using oligo(dT) primer (IDT) and SuperScript II RT (Invitrogen). For qPCR analysis, 2 ul of prepared cDNA was mixed with iTAQ universal Syber Green supermix (Invitrogen) and 300uM of ERAAP primers. ERAAP mRNA levels were compared by ΔΔCT using average Cq values normalized to GAPDH.

#### Spleen and liver cell isolation

Spleen cells from mice were homogenized in PBS using a GentleMACS Dissociator (Miltenyi Biotec), washed in FACS buffer (PBS+0.1%sodium azide+5% FCS), and passed through a 70um filter. Cells were RBC lysed with ACK lysis buffer (0.15M NH_4_CL, 1mM KHCO_3_, 0.1mM Na_2_EDTA). Livers were perfused with PBS and homogenized in PBS using a GentleMACS Tissue Dissociator (Miltenyi Biotec). Liver samples were then incubated with 0.1% collagenase type 1A and 40 ug/mL of DNase I for 20 minutes at 37°C before washing with cRPMI and passing through a 70uM filter. Lymphocytes were isolated using a Percoll gradient of 40% percoll layer overlaid onto a 70% percoll layer after spinning at 500xg for 30 minutes at room temperature.

#### Antibodies and flow cytometry

Antibody staining was done in 2.4G2 media with cell surface antibodies for 30 min on ice. The following antibodies and clones were used for staining: CD8a Clone 53-6.7, B220 Clone RA3-6B2, Va3.2 Clone RR3-16, KLRG1 Clone 2F1/KLRG1, CD44 Clone IM7, CD8b Clone YTS156.7.7, CD45.1 Clone A20, and IFNy Clone XMG1.2.

#### *Ex vivo* T cell restimulation assay

Spleens were harvested from mice 10 days post-immunization or infection, and 5e6 spleen cells were stimulated *in vitro* with 5e6 irradiated ERAAP KO spleen cells and 20 U/ml of recombinant human IL-2 (BD Biosciences). Human IL-2 works the same as mouse IL-2 and was used for convenience and cost. After 6 days, cells were isolated and restimulated for 5 hours with CD4 and CD8-depleted WT or ERAAP KO spleen cells. Golgi-Plug (BD Biosciences) was added after 4 hours of re-stimulation. Cells were stained with surface markers, fixed, permeabilized (Cytofix/Cytoperm Kit BD Biosciences), and stained intracellularly for IFN-γ.

#### *In vivo* cytotoxicity assay

WT mice were infected with 1e6 PFU of WT MCMV intraperitoneally. For controls, mice were immunized with 2e7 of WT or ERAAP KO spleen cells intraperitoneally. WT and ERAAP KO spleen cells were isolated and stained with 0.5uM or 5uM of CFSE dye (ThermoFisher), respectively. Cells were counted and 5e6 each of WT (CFSE^lo^) and ERAAP KO (CFSE^hi^) were mixed and injected intravenously into immunized and infected mice. After 24 hours, spleen cells from each mouse were isolated, and the ratio of CFSE^lo^ (WT cells) to CFSE^hi^ (ERAAP-KO cells) was measured by flow cytometry. Percent killing was calculated as follows: 1-(%CFSE^hi^ sample / %CFSE^lo^ sample) / (%CFSE^hi^ uninfected / %CFSE^lo^ uninfected) x 100.

#### *In vivo* antigen presentation assay

WT B6 mice were infected with 1e6 PFU of MCMV intraperitoneally. Two days later, QFL and OT-1 transgenic T cells were labeled with 5uM CFSE or 5uM eF670, respectively. 1e6 labeled QFL cells and 1e6 labelled OT-1 cells were mixed 1:1 and injected intravenously into infected mice. After 4 days, the liver and spleen were isolated and processed, and proliferation was analyzed by flow cytometry.

#### Adoptive transfer of T cells and MCMV infection in Rag2/γc knockout mice

1e4, 2.5e5, or 1e6 QFL or OT-1 T cells from TCR transgenic mice were adoptively transferred intravenously into Rag2/γc knockout mice. After 2 days post-transfer, mice were infected intraperitoneally with 1e6 PFU of MCMV. After 12 days post-infection, the liver and spleen were isolated from infected mice and homogenized. MCMV titers were determined by qPCR in both the spleen and liver.

#### QFL T cell enrichment

QFL T cells were enriched using a QFL tetramer (FL9-Qa-1b) synthesized by the NIH tetramer core facility. Spleen cells were isolated as stated above and incubated with 50 nM of Dasatinib (Cell signaling #9052) at 37°C for 30 minutes. Cells were then washed and incubated with PE-QFL tetramer (1:200) for 1 hour at RT. Cells were washed, resuspended in 150ul FACS buffer with 100 ul of anti-PE microbeads (Miltenyi Biotec), and incubated for 20 minutes at 4°C. Cells were then washed and passed through an LS magnetic column (Miltenyi Biotec). Enriched cells were stained with antibodies for B220, CD8α, CD8β, and Vα3.2. Tetramer+ QFL T cells were gated as B220^−^ CD8α^+^QFL Tet^+^Vα3.2^+^. CountBright beads (Invitrogen) were used in each sample to measure total cell numbers in the enriched and unenriched samples.

### QUANTIFICATION AND STATISTICAL ANALYSIS

Statistical analysis for each experiment was done with Prism 7 (Graphpad Software) using ordinary one-way, two-way ANOVA multiple comparisons tests, or Mann-Whitney t-test. Compiled data is shown as mean±SEM. (ns=not significant, *p<0.0332, **p<0.0021, ***p<0.0002, ****p<0.0001).

## Supplementary Material

1

## Figures and Tables

**Table:** KEY RESOURCES TABLE

REAGENT or RESOURCE	SOURCE	IDENTIFIER
Antibodies		
Anti-mouse CD8a (53-6.7)	BioLegend	Cat#: 100743; RRID: AB_2561352
Anti-mouse CD45R/B220 (RA3-6B2)	BD Biosciences	Cat#: 561102; RRID: AB_394335
Anti-mouse TCR Va3.2 (RR3-16)	eBioscience	Cat#: 11-5799-80; RRID: AB_2572504
Anti-mouse KLRG1 (2F1)	eBioscience	Cat#: 25-5893-82; RRID: AB_1518768
Anti-mouse CD44 (IM7)	eBioscience	Cat#: 17-0441-82; RRID: AB_469390
Anti-mouse CD8b (YTS156.7.7)	BioLegend	Cat#: 126615; RRID: AB_2562776
Anti-mouse CD45.1 (A20)	BD Biosciences	Cat#: 560520; RRID: AB_1727490
Anti-mouse IFN gamma (XMG1.2)	eBioscience	Cat#: 61-7311-82; RRID: AB_2574662
Bacterial and virus strains		
WT MCMV	VR-194 (Smith) strain of mCMV was obtained from The American Type Culture Collection	N/A
MCMV-GFP	Generous gift from the Hamilton lab (Duke University, Durham, NC) (Henry et al., 2000^81^)	N/A
MHV68	Obtained from the laboratory of Sam Speck (The Emory Vaccine Center, Atlanta, Georgia)	N/A
Chemicals, peptides, and recombinant proteins		
Ghost Dye Violet 510	Tonbo Biosciences	Cat#: 13-0870-T500
CellTrace^™^ CFSE Cell Proliferation Kit	ThermoFisher	Cat#: C34554
Recombinant Human IL-2	BD Biosciences	Cat#: 554603
Dasatinib	Cell Signaling Techology	Cat#: 9052S
FL9-Qa-1b monomer	NIH Tetramer Core Facility	N/A
Critical commercial assays		
Dynabeads^™^ Sheep-Anti Mouse IgG	ThermoFisher	Cat#: 11031
Anti-PE MicroBeads	Miltenyi Biotec	Cat#: 130-048-801
Anti-PE MicroBeads	Miltenyi Biotec	Cat#: 130-090-855
CountBright^™^ Absolute Counting Beads	ThermoFisher	Cat#: C36950
Fixation/Permeabilization Solution Kit with BD GolgiPlug^™^	BD Biosciences	Cat#: 555028
Experimental models: Cell lines		
NIH 3T3	ATCC	CRL-1658
BEko8Z	Nagarajan et al., 2012^28^	N/A
RAW 264.7	ATCC	TIB-71
B6 fibroblasts	Lander et al., 1978^82^	N/A
Experimental models: Organisms/strains		
Rag2/Il2rg Double Knockout	Taconic	Cat#: 4111-M
Rag2/OT-I	Taconic	Cat#: 2334-M
C57BL/6J	The Jackson Laboratory	Cat#: 000644
QFL TCR Transgenic	Cancer Research Laboratory Gene Targeting Facility at UC Berkeley	N/A
ERAAP-deficient mice (ERAAP KO)	Hammer et al., 2006^83^	N/A
Qa-1b-deficient mice (Qa-1b KO)	Hu et al., 2004^84^	N/A
MCMV gB qPCR Forward Primer	Khairallah et al., 2015^85^ F: AGGCCGGTCGAGTACTTCTT	N/A
MCMV gB qPCR Reverse Primer	Khairallah et al., 2015^85^ R: GCGCGGAGTATCAATAGAGC	N/A
GAPDH qPCR Forward Primer	F: GAAGGTCGGTGTGAACGGA	N/A
GAPDH qPCR Reverse Primer	R: GTTAGTGGGGTCTCGCTCCT	N/A
Software and algorithms		
FlowJo	FlowJo LLC	Version 10
Prism (Version 7)	GraphPad Software	N/A

## Highlights

- MCMV downregulates ERAAP, leading to FL9 presentation on Qa-1b
- MCMV infection induces non-classical, Qa-1b-restricted QFL T cells to proliferate
- QFL T cells acquire an effector phenotype and protect against severe MCMV infection

## INCLUSION AND DIVERSITY

We support inclusive, diverse, and equitable conduct of research. We worked to ensure sex balance in the selection of non-human subjects. We worked to ensure diversity in experimental samples through the selection of the cell lines. One or more of the authors of this paper self-identifies as an underrepresented ethnic minority in their field of research or within their geographical location. One or more of the authors of this paper self-identifies as a member of the LGBTQIA+ community.
