# Supplementary material for: Murine cytomegalovirus downregulates ERAAP and induces an unconventional T cell response to self
Source: Cell Rep. Author manuscript; Available in PMC 2023 Oct 23. (PMC10539480; doi:10.1016/j.celrep.2023.112317)
Supplement: 1 [file NIHMS1898012-supplement-1.pdf]

Cell Reports, Volume *42*

Supplemental information

Murine cytomegalovirus downregulates ERAAP and induces an unconventional T cell response to self

Kristina M. Geiger, Michael Manoharan, Rachel Coombs, Kathya Arana, Chan-Su Park, Angus Y. Lee, Nilabh Shastri, Ellen A. Robey, and Laurent Coscoy

Supplemental Figure 1

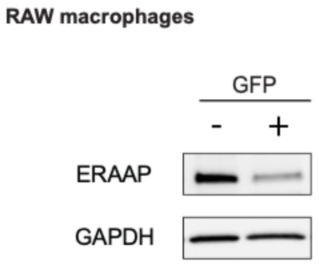

Supplemental Figure 1 (Related to Figure 1): ERAAP downregulation in MCMV infected RAW macrophages

Representative western blot showing ERAAP protein levels in GFP positive (+) and GFP negative (-) samples, with GAPDH as the loading control from two independent experiments. RAW macrophages were infected with MCMV-GFP (MOI 10) and after 36 hours GFP+ and GFP- cells were sorted and assayed by western blot.

Supplemental Figure 2

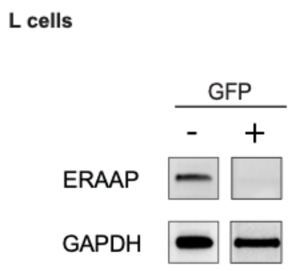

Supplemental Figure 2 (Related to Figure 1): ERAAP downregulation in MCMV infected L cells
Representative western blot showing ERAAP protein levels in GFP positive (+) and GFP negative (-) samples, with GAPDH as the loading control from two independent experiments. L cells were infected with MCMV-GFP (MOI 10) and after 36 hours GFP+ and GFP- cells were sorted and assayed by western blot.

Supplemental Figure 3

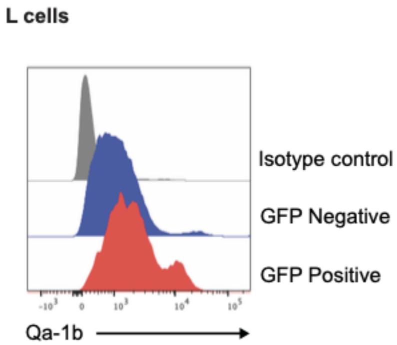

Supplemental Figure 3 (Related to Figure 1): Qa-1b levels remain unchanged in MCMV infected L cells
Representative flow cytometry plots showing surface expression of non-classical MHC Qa-1b in GFP- (blue) and GFP+ (red) MCMV infected L cells (MOI 10) from two independent experiments. Gray histograms represent isotype antibody control.

Supplemental Figure 4: Model for ERAAP downregulation and QFL activation during MCMV infection

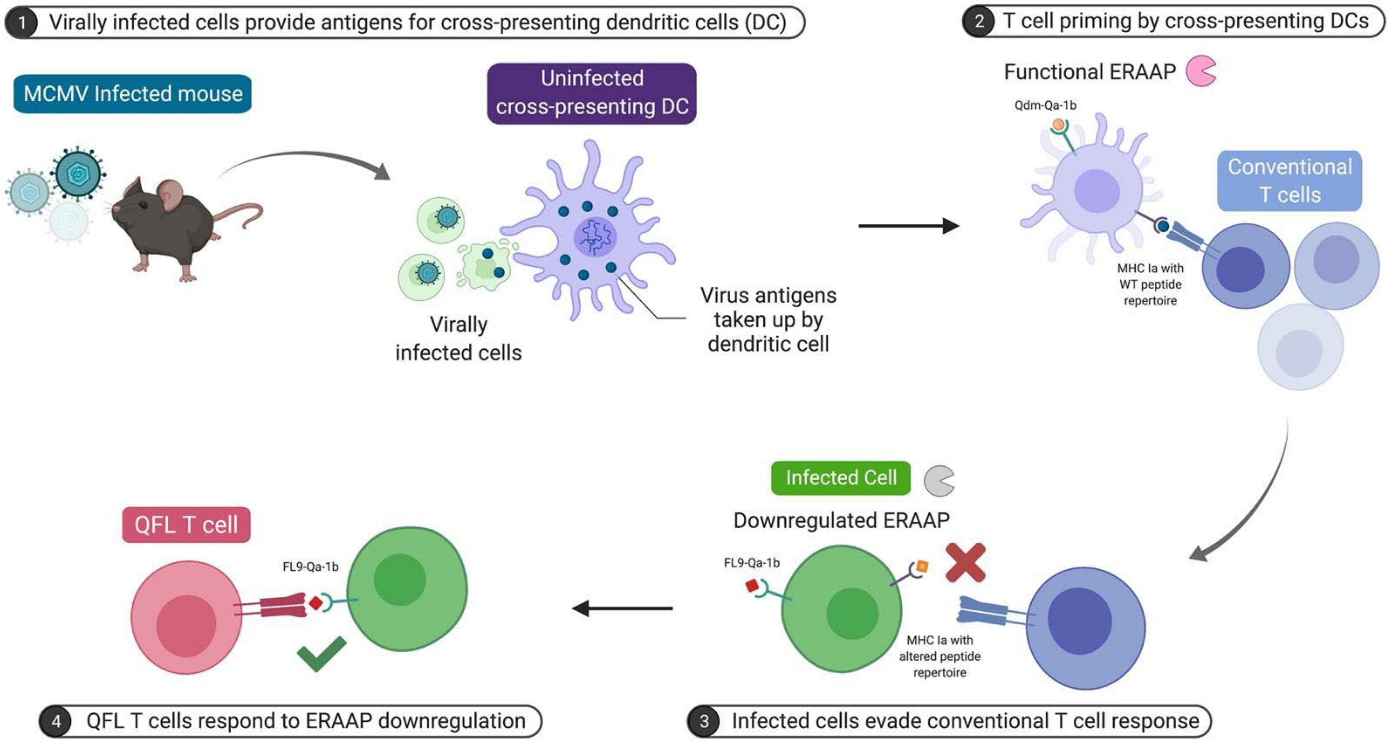

Supplemental Figure 4: Model for ERAAP downregulation and QFL activation during MCMV infection
(1-3) During MCMV infection, cross-presenting dendritic cells (DCs) with functional ERAAP and MHC I prime conventional $CD8^+$ T cell responses against MCMV. We hypothesize that MCMV infected cells with downregulated ERAAP present an altered peptide repertoire to partially evade conventional T cell responses. ERAAP downregulation may also lead to decreased presentation of viral peptides, and ones that are presented may have a lower affinity. As a result, we propose that ERAAP downregulation serves as an alternate way for MCMV to partially evade conventional T cell responses and lower the efficiency of responses that do recognize infected cells. (4) However, by downregulating ERAAP, this leads to the presentation of FL9 on Qa-1b, thus inducing QFL T cells to expand in response to this ligand. As a result, these QFL T cells recognize and target infected cells and play an anti-viral role during MCMV infection.
